# Effect of genotype imputation on genome-enabled prediction of complex traits: an empirical study with mice data

**DOI:** 10.1186/s12863-014-0149-9

**Published:** 2014-12-29

**Authors:** Vivian PS Felipe, Hayrettin Okut, Daniel Gianola, Martinho A Silva, Guilherme JM Rosa

**Affiliations:** Department of Animal Sciences, University of Wisconsin, Madison, 53706 USA; Department of Animal Sciences, Biometry and Genetics Branch, University of Yuzuncu Yil, Van, 65080 Turkey; Department of Animal Sciences, Federal University of Jequitinhonha and Mucuri Valleys, Minas Gerais, Brazil

**Keywords:** Genotype imputation, Genome-enabled prediction, Complex traits, Non-linear models

## Abstract

**Background:**

Genotype imputation is an important tool for whole-genome prediction as it allows cost reduction of individual genotyping. However, benefits of genotype imputation have been evaluated mostly for linear additive genetic models. In this study we investigated the impact of employing imputed genotypes when using more elaborated models of phenotype prediction. Our hypothesis was that such models would be able to track genetic signals using the observed genotypes only, with no additional information to be gained from imputed genotypes.

**Results:**

For the present study, an outbred mice population containing 1,904 individuals and genotypes for 1,809 pre-selected markers was used. The effect of imputation was evaluated for a linear model (the Bayesian LASSO - BL) and for semi and non-parametric models (Reproducing Kernel Hilbert spaces regressions – RKHS, and Bayesian Regularized Artificial Neural Networks – BRANN, respectively). The RKHS method had the best predictive accuracy. Genotype imputation had a similar impact on the effectiveness of BL and RKHS. BRANN predictions were, apparently, more sensitive to imputation errors. In scenarios where the masking rates were 75% and 50%, the genotype imputation was not beneficial. However, genotype imputation incorporated information about important markers and improved predictive ability, especially for body mass index (BMI), when genotype information was sparse (90% masking), and for body weight (BW) when the reference sample for imputation was weakly related to the target population.

**Conclusions:**

In conclusion, genotype imputation is not always helpful for phenotype prediction, and so it should be considered in a case-by-case basis. In summary, factors that can affect the usefulness of genotype imputation for prediction of yet-to-be observed traits are: the imputation accuracy itself, the structure of the population, the genetic architecture of the target trait and also the model used for phenotype prediction.

## Background

Genome-enabled prediction of quantitative traits is a topic of current interest in genetic improvement of agricultural animal and plant species, as well as in preventive and personalized medicine in humans. In agriculture, it has been applied to prediction of genetic merit for breeding purposes [1] and to management decisions based on predicted phenotypes [2,3]. In human medicine, it has been applied for example to prediction of risk to disease [4,5]. The original idea was proposed by Meuwissen et al. [6] and involves the use of prediction models including thousands of Single Nucleotide Polymorphisms (SNPs) fitted simultaneously as predictor variables, generally using shrinkage-based estimation techniques (e.g. [7]). The implementation of such models involves two steps. First, a group of individuals having both phenotypic and genotypic information (generally referred to as reference sample) is used to train the model. Cross-validation techniques can be used to compare different models. Secondly, the trained model is applied to a group of individuals with genotypic information only (the target sample), for prediction of their genetic merit or of their yet-to-be-observed phenotypes.

A commonly used technique in this field is genotype imputation. Genotype imputation can be employed to fill in missing data from the laboratory or allow merging data sets generated from different SNP chips. Genotype imputation has been proposed also to impute from genotypes scored with low-density chips to higher densities, as a way to reduce genotyping costs [3,8,9]. Other authors have proposed to use cosegregation information from chips built with evenly spaced low-density SNPs or SNPs selected by their estimated effects to track signals of high density SNP alleles [10]. Weigel et al. [11] showed that a low-density panel containing selected SNPs can retain most of the prediction ability of high-density panels. Furthermore, in a later study, Weigel et al. [3] also showed that imputed genotypes can provide similar levels of predictive ability to those derived from high density genotypes in scenarios where a suitable reference population is available.

The benefit of imputing genotypes essentially depends on its imputation accuracy [3], which, in turn, depends on a number of factors including population structure [3,12], and genetic architecture of the target trait [13]. Many studies have shown that currently available imputation methods and software give a satisfactory level of accuracy of uncovering unknown genotypes [8,14-16]. Hence, imputation may provide a suitable alternative for reducing genotyping costs, and it has been suggested for commercial applications such as the pre-screening of young bulls and heifers in dairy cattle [3]. Moreover, VanRaden et al. [17] reported that the reliability of genomic predictions can be improved at a lower cost by combining information from chips containing varied marker densities, to increase both the number of markers and animals included in genome-based evaluation.

So far, all studies conducted to evaluate the effect of genotype imputation on whole-genome prediction have assumed a linear relationship between phenotype and genotype, aimed at capturing additive genetic effects only. However, complex traits are known to be affected by complex gene effects and interactions [18]. For this reason, interest in non- and semi-parametric methods for prediction of complex traits using genomic information has been increasing. Such methods include Reproducing Kernel Hilbert Spaces (RKHS) regressions on markers [19-21] radial basis functions [22,23], and artificial neural networks [24,25]. Gianola et al. [24] argued that these non-parametric regressions can capture complex interactions and nonlinearities, which is not possible with Bayesian linear regressions commonly used in genomic prediction.

Recently, Heslot et al. [26] evaluated the prediction accuracy of several models including Bayesian regression methods and machine learning techniques. Their results indicated a slight superiority of non-linear models for phenotype prediction in plants. As another example, Okut et al. [25] used Bayesian Regularized Neural Networks (BRANN) to predict body mass index (BMI) in mice using information on 798 SNPs, and obtained an overall correlation between observed and predicted data that varied between 0.25 and 0.3. Similar results were obtained by de los Campos et al. [27] using a Bayesian LASSO approach but using a panel that was 13 times larger, comprising 10,946 SNPs. Perez-Rodriguez et al. [28] compared linear and nonlinear models for genome-enabled prediction in wheat and showed that nonlinear models in general performed better. However, the author found that in this case the BRANN did not outperformed the BL. Lastly, Howard et al. [29] indicated a clear superiority of RKHS when predicting epistatic traits using simulation.

The objective of our study was to investigate the effect of genotype imputation in the context of whole-genome prediction of complex traits in mice using parametric, semi-parametric and non-parametric models applied to different sizes of subsets of SNPs. Our underlying hypothesis was that more elaborated prediction models, such as those capable to accommodate non-additive genetic effects, would not benefit significantly from genotype imputation for prediction of yet-to-be-observed phenotypes.

## Results

Results indicated a good accuracy of imputation of unknown genotypes for all scenarios (Table 1). The lowest imputation accuracy (0.75) was for the scenario with approximately 90% of the genotypes masked and the reference panel was not related to the imputing set. Although Beagle software does not use pedigree information, a higher genetic relatedness among individuals in the reference panel and in the set containing missing genotypes can enhance imputation accuracy. The explanation is that similarity of linkage disequilibrium (LD) patterns between the set to be imputed and the reference panel serves as a basis for imputing the unknown genotypes. The most common error found was the switch between heterozygotes and homozygotes for the allele at higher frequency (about 65%).

**Table 1:** **Overall imputation accuracy and error distribution for 90**, **75 and 50**% **of masked genotypes**

	**90%**		**75%**		**50%**	
	**Across families**	**Within families**	**Across families**	**Within families**	**Across families**	**Within families**
Accuracy	0.75	0.79	0.91	0.94	0.97	0.98
0*<−>1* error^a^	0.16	0.17	0.22	0.25	0.26	0.20
1<−>2* error^b^	0.50	0.54	0.61	0.63	0.62	0.65
0<−>2 error^c^	0.09	0.08	0.08	0.06	0.09	0.13

^a^Error due to change from 0 to 1 genotype code or vice versa.

^b^Error due to change from 1 to 2 genotype code or vice versa.

^c^Error due to change from 0 to 2 genotype code or vice versa.

*Genotypes are coded as 0, 1 and 2 as the number of copies of the more frequent allele.

Correlations between predicted and observed phenotypes in the testing set are shown in Tables 2 and 3 for body weight (BW) and body mass index (BMI), respectively. The distribution of individuals into training and testing sets affected the predictive ability of all models considered. A higher genetic relatedness between these two sets provided better prediction accuracy for BW. On the other hand, for BMI, the average correlation between predicted and observed phenotypes was higher for the across families layout. Therefore, information from closely related individuals for SNP effect estimation was beneficial for prediction of new phenotypes, at least for BW.

**Table 2:** **Correlations between predicted and observed body weight for all masking rates and family layouts**

**90%** **genotype masking rate**						
Model*	**Across families**			**Within families**		
	1809	1809i^a^	201	1809	1809i^a^	201
BL	0.347	0.259	0.169	0.500	0.330	0.407
RKHS	0.347	0.312	0.210	0.527	0.417	0.499
BRANN	0.330	0.217	0.144	0.490	0.274	0.392
**75%** **genotype masking rate**						
Model*	**Across families**			**Within families**		
	1809	1809i^b^	453	1809	1809i^b^	453
BL	0.343	0.291	0.262	0.499	0.447	0.430
RKHS	0.348	0.317	0.293	0.528	0.506	0.501
BRANN	0.320	0.241	0.255	0.492	0.414	0.428
**50%** **genotype masking rate**						
Model*	**Across families**			**Within families**		
	1809	1809i^c^	905	1809	1809i^c^	905
BL	0.342	0.324	0.271	0.499	0.496	0.477
RKHS	0.343	0.345	0.306	0.530	0.530	0.520
BRANN	0.320	0.281	0.252	0.492	0.478	0.461

^a^Imputed from 201 SNPs.

^b^Imputed from 453 SNPs.

^c^Imputed from 905 SNPs.

*BL: Bayesian LASSO; RKHS: Reproducing Kernel Hilbert Spaces (RKHS) and; BRANN: Bayesian Regularized Neural Networks.

**Table 3:** **Correlations between predicted and observed body mass index for all genotype masking rates and family layouts**

**90%** **genotype masking rate**						
Model*	**Across families**			**Within families**		
	1809	1809i^a^	201	1809	1809i^a^	201
BL	0.227	0.193	0.191	0.199	0.164	−0.047
RKHS	0.238	0.195	0.199	0.208	0.132	−0.054
BRANN	0.112	0.092	0.147	0.163	0.041	0.054
**75%** **genotype masking rate**						
Model*	**Across families**			**Within families**		
	1809	1809i^b^	453	1809	1809i^b^	453
BL	0.228	0.219	0.199	0.200	0.196	0.184
RKHS	0.238	0.226	0.211	0.208	0.204	0.200
BRANN	0.118	0.115	0.145	0.172	0.154	0.170
**50%** **genotype masking rate**						
Model*	**Across families**			**Within families**		
	1809	1809i^c^	905	1809	1809i^c^	905
BL	0.227	0.231	0.225	0.199	0.197	0.189
RKHS	0.238	0.238	0.236	0.207	0.206	0.202
BRANN	0.118	0.131	0.149	0.172	0.168	0.149

^a^Imputed from 201 SNPs.

^b^Imputed from 453 SNPs.

^c^Imputed from 905 SNPs.

*BL: Bayesian LASSO; RKHS: Reproducing Kernel Hilbert Spaces (RKHS) and; BRANN: Bayesian Regularized Neural Networks.

As expected, the predictive ability for BW was higher than for BMI, since the latter has a lower heritability. Differences on results for each trait are also probably due to differences between their underlying genetic architectures. As discussed by Legarra et al. [30], in this data set there is some confounding between family and cage effects since most animals allocated to the same cage were full sibs, so it is possible that the additive genetic effect is understated. For the present study however, it is reasonable to assume that this issue would impact the predictive ability of the different models considered in a similar way.

In general, the method with the best prediction results was RKHS using kernel averaging, and the worst was BRANN, probably due to overfitting. BRANN showed high correlation (above 0.9) between predicted and measured phenotype for the training sets (results not shown). Table 2, which describes results for BW, shows that imputation seemed to be beneficial for phenotype prediction when relatedness between reference and target samples was poorer, especially for BL and RKHS. Table 3, in contrast, shows a markedly noticeable benefit of imputation when the number of markers available in the testing set was low (201 SNPs) for the within-family layout when predicting BMI. Regarding the methods, imputation seemed to have similar impact on efficiency of BL and RKHS, whereas for BRANN it resulted in less robust predictions due to imputation error. In scenarios with good imputation accuracy and masking rates of 75% and 50%, the genotype imputation did not bring great benefit, as seen in Tables 2 and 3. However, when genotype information was sparse (90% masking rate – 201 observed genotypes) imputation could bring information about important markers to improve phenotypic prediction.

The results for predicted mean squared error (PMSE) are summarized in Tables 4 and 5 for BW and BMI, respectively. For BW, the lowest values of PMSE were found for predictions made within families with the full data set (1,809 SNPs). This agrees with the results obtained for predictive correlation described earlier. In general, higher masking rates resulted in a higher PMSE for BW and data containing imputed genotypes provided a better goodness of fit compared to the data with no genotype imputation when markers were masked. With BMI, however, the PMSE showed no changes according to genotype masking rates or genotype imputation for BL and RKHS models. Overall, BRANN had the highest PMSE values, in agreement with the results using correlation between observed and predicted phenotypes.

**Table 4:** **Prediction mean squared errors for body weight analysis by family layouts and genotype masking rates**

**90%** **genotype masking rate**						
Model*	**Across families**			**Within families**		
	1809	1809i^a^	201	1809	1809i^a^	201
BL	5.03	5.32	5.67	4.18	4.99	4.71
RKHS	4.92	5.20	5.36	4.15	4.75	4.66
BRANN	5.36	5.52	5.54	5.26	5.40	5.52
**75%** **genotype masking rate**						
Model*	**Across families**			**Within families**		
	1809	1809i^b^	453	1809	1809i^b^	453
BL	5.05	5.25	5.44	4.18	4.45	4.52
RKHS	4.92	5.04	5.11	4.13	4.23	4.21
BRANN	5.38	5.44	5.44	5.26	5.32	5.33
**50%** **genotype masking rate**						
Model*	**Across families**			**Within families**		
	1809	1809i^c^	905	1809	1809i^c^	905
BL	5.06	5.12	5.49	4.18	4.19	4.32
RKHS	4.94	4.94	5.01	4.06	4.08	4.12
BRANN	5.20	5.24	5.44	5.26	5.27	5.28

^a^Imputed from 201 SNPs.

^b^Imputed from 453 SNPs.

^c^Imputed from 905 SNPs.

*BL: Bayesian LASSO; RKHS: Reproducing Kernel Hilbert Spaces (RKHS) and; BRANN: Bayesian Regularized Neural Networks.

**Table 5:** **Prediction mean squared errors for body mass index analysis by family layouts and genotype masking rates**

**90%** **genotype masking rate**						
Model*	**Across families**			**Within families**		
	1809	1809i^a^	201	1809	1809i^a^	201
BL	0.002	0.002	0.002	0.002	0.002	0.002
RKHS	0.002	0.002	0.002	0.002	0.002	0.002
BRANN	0.013	0.014	0.010	0.042	0.036	0.024
**75%** **genotype masking rate**						
Model*	**Across families**			**Within families**		
	1809	1809i^b^	453	1809	1809i^b^	453
BL	0.002	0.002	0.002	0.002	0.002	0.002
RKHS	0.002	0.002	0.002	0.002	0.002	0.002
BRANN	0.021	0.023	0.015	0.044	0.045	0.041
**50%** **genotype masking rate**						
Model*	**Across families**			**Within families**		
	1809	1809i^c^	905	1809	1809i^c^	905
BL	0.002	0.002	0.002	0.002	0.002	0.002
RKHS	0.002	0.002	0.002	0.002	0.002	0.002
BRANN	0.021	0.023	0.016	0.040	0.040	0.047

^a^Imputed from 201 SNPs.

^b^Imputed from 453 SNPs.

^c^Imputed from 905 SNPs.

*BL: Bayesian LASSO; RKHS: Reproducing Kernel Hilbert Spaces (RKHS) and; BRANN: Bayesian Regularized Neural Networks.

## Discussion

Recently, some studies have investigated the predictive ability of models using subsets of SNPs, with and without imputation [8,31,32]. In general, predictive ability improved with imputed genotypes, such that many researchers recommend this strategy to decrease costs on genomic selection programs. However, most studies with genotype imputation in whole-genome predictions considered only linear models, such as ridge regression, Bayesian LASSO or GBLUP approaches [3,8,12] specifically suited to model additive genetic signals but not tailored to capture non-additive genetic effects such as dominance and epistasis. The goal of our study was to explore if more elaborated models, such as semi-parametric and non-parametric methods, could track genetic signals from low-density chips without the need of imputing to higher density chips.

The results obtained indicated that imputation of the missing genotypes was not always advantageous for phenotypic prediction. The benefit of imputing genotypes depended on the degree of relatedness between reference and target samples, genetic architecture of the trait, number of markers available in the original panel, and the method used to predict marker effects.

Weigel et al. [3] investigated the effect of imputation from a low-density chip to a 50K chip on the accuracy of direct genomic values in Jersey cattle using BL. They found that genotype imputation improved predictive ability in scenarios where imputation accuracy was high; otherwise, a reduced panel containing the original number of SNPs was preferred. In the same context, Mulder et al. [8] showed that due to the magnitude of imputation errors, the noise added by imputation can be greater than its benefit when predicting breeding values. Hence, only those SNPs with high imputation accuracy would have a positive effect on the reliability of direct genomic value predictions. In the present study, results also suggested that if imputation accuracy was low, the model containing only observed marker genotypes gave a better prediction than the imputed set. The correlation between predicted and measured BW within families using either a full data set containing 1,809 genotyped SNPs, or the full data set containing 90% imputed genotypes, or a reduced panel of marker genotypes (201 SNPs) was respectively 0.52, 0.42 and 0.50 using RKHS. This indicates that imputation brought no additional information to the model.

For scenarios with different masking rates the imputed testing set gave, on average, a 4% higher correlation. For BMI, the reduced testing sets (201, 453 or 905 SNPs) provided 89% of the predictive ability of their respective complete imputed testing sets and 78% of the predictive ability of the complete testing sets, averaged across all scenarios tested. So, in general, the results indicated that imputation can be useful for phenotypic prediction.

When comparing correlations for across and within families cross-validation strategies, genotype imputation seemed to be more effective in improving prediction accuracy in cases where there was a weaker genetic relationship among individuals in the reference and testing data sets. Other studies regarding the role of within and across-family information [30] also indicate the need of genotyping and phenotyping closely related individuals, in order to improve predictive ability. As such, this information is an important issue for designing genome-assisted breeding programs.

Regarding the models considered, it was expected that the non-parametric methods would give smaller differences between the complete set with imputed markers and the reduced panel. However, our results indicated that the effect of imputation was similar for BL and RKHS predictions. An exception was the case of BRANN, which was not able to cope with imputation errors and tended to give worse predictions for the complete testing set containing imputed markers. Therefore, it seems that imputation accuracy is a fundamental factor to be considered when using BRANN for predicting phenotypes. The imputation from 905 markers to the full panel (1,809 SNPs) tended to slightly improve prediction using BRANN perhaps due to the low imputation errors rates for these panels.

Another discussion, beyond the scope of this paper, is on differences between chips containing either equally spaced SNPs or SNPs pre-selected based on their estimated effects for genome-enabled prediction (e.g., [33]). The main advantage of the former is that it avoids the need of trait-specific low-density SNP panels and, in general, it has given reliability of genomic breeding values similar to the latter [13]. Comparing the results obtained with the available literature on genomic selection applied to this same data set, it was found that no important differences in predictive ability were observed when using the entire set of SNPs. For example, de los Campos [27] used 10,946 SNPs with a BL model and observed a rank correlation of 0.306 between phenotypic observations and genomic predictions for BMI. Here, we obtained almost 95% of this correlation using the same method but with only 1,809 evenly spaced SNPs. In addition, Okut et al. [25] reported a correlation between predictions and observations in the testing set of 0.18 for BMI using BRANN and 798 pre-selected markers. We obtained a correlation of 0.15 with the same model and 905 evenly spaced markers, which suggests that BRANN can work better using selected markers with larger effects.

Similar results were observed in terms of PMSE. Apparently, higher imputation errors caused higher values of PMSE, making the results from models using the reduced SNP panel better than those containing imputed marker genotypes.

The results of the present study can be generalized for different scenarios, regardless the number of SNPs and/or sample size of a particular study, based on the impact of imputation accuracy on the predictive quality of genomic models. Clearly, the predictive ability of a model not only depends on how well genotypes are imputed but also on the genetic architecture of the target trait and the breeding program design. Therefore, the general reasoning provided by the results of the present study is that the use of genotype imputation should the evaluated in a case-by-case basis. For example, the use of imputed genotypes when employing the non-parametric method (BRANN, in this case) is not recommended given that this model tends to approximate the noise inserted by imputation errors.

## Conclusions

Genotype imputation did not always improve the predictive ability of parametric and semi-parametric models. For BW, genotype imputation improved predictive ability when there was a relatively low genetic relatedness between the reference panel and the target population set. For BMI, the use of genotype imputation was more beneficial when the genotype set was very sparse (201 SNPs), especially for BL and RKHS. In other scenarios, imputation just slightly improved or even deteriorated predictive ability; the latter happened in cases in which the genotype imputation had low accuracy. Lastly, BRANN seemed more sensitive to imputation errors; therefore the use of imputed genotypes with this model should be carefully evaluated when using neural networks.

## Methods

### Data

A publicly available dataset on mice (http://mus.well.ox.ac.uk/mouse/HS/) was used. This is a sample from an outbred mice population that descended from eight inbred strains created for fine-mapping QTL and high-resolution whole-genome association analysis of quantitative traits [34]. The data set contains genotypic information from 1,904 fully pedigreed mice on 13,459 SNPs coded as 0, 1 and 2 as the number of copies of the more frequent allele. Traits such as weight, immunology, obesity and behavior, to name a few, are also available for a proportion of these animals. A full description of this mice population is in [35] and [36]. This data have also been utilized in genomic-enabled prediction studies using Bayesian regression methods [2,27,30,37] and neural networks [25].

In our analysis, only animals with both phenotypic and genotypic information were considered. Loci with a minor allele frequency lower than 0.05, a call rate lower than 95% or not in Hardy-Weinberg equilibrium (p<0.01) were discarded from the original dataset. The two traits, BW at ten weeks of age, and body mass index BMI were pre-corrected by fitting the following linear mixed model:

$$ \boldsymbol{y}=\boldsymbol{X}\boldsymbol{\theta } +\boldsymbol{W}\boldsymbol{c}+\boldsymbol{Z}\boldsymbol{u}+\boldsymbol{e}, $$

where *y* is the vector of observations on one of the measured phenotypes (BW or BMI); *θ* is an unknown vector of fixed effects of age, gender, month and cage density; *c* is a random vector of unknown cage effects; *u* is a random vector of unknown additive genetic effects; ***X***, ***W*** and ***Z*** are the incidence matrices of fixed, random cage and additive genetic effects, respectively, and *e* is a vector of residual effects assumed to follow a multivariate normal distribution $\boldsymbol{e}\sim N\left(\mathbf{0},\boldsymbol{I}{\boldsymbol{\sigma}}_e^{\mathbf{2}}\right)$, where ${\sigma}_e^2$ is the residual variance. The random additive genetic and cage effects were assumed independent from each other and with distributions $\boldsymbol{u}\sim N\left(\mathbf{0},\boldsymbol{A}{\boldsymbol{\sigma}}_{\mathrm{u}}^2\right)$ and $\boldsymbol{c}\sim N\left(\mathbf{0},\boldsymbol{I}{\boldsymbol{\sigma}}_{\mathrm{c}}^2\right)$, respectively, where A is the additive genetic relationship matrix, I is an identity matrix of appropriate order, and ${\upsigma}_{\mathrm{u}}^2$ and ${\upsigma}_{\mathrm{c}}^2$ are additive genetic and cage components of variance, respectively. The target response variable after correction was ${\mathbf{y}}^{*}=\mathbf{y}-\mathbf{X}\widehat{\boldsymbol{\uptheta}}-\mathbf{W}\widehat{\mathbf{c}}$, which presumably includes all types of genetic effects (additive, dominance and epistasis) as well as additional environmental effects not accounted for by the mixed model employed. From now on the pre-corrected phenotype y* will be simply referred to as **y**.

After data cleaning, 10,348 SNPs remained from which 1,809 equally spaced SNPs were selected and regarded as full genotyped data due to computational limitations on number of markers that can be fitted when using Bayesian Regularized Neural Networks. Then, subsets containing 905, 453 and 201 (50, 75 and 90% masking rates, respectively) equally spaced SNPs were taken from the full genotype set. In total, 1,881 and 1,823 individuals were included in the analysis of BW and BMI, respectively. For a cross-validation (CV) model comparison, in each case, approximately 2/3 of the individuals were designated as training set (reference sample) and 1/3 as testing set (target sample) (See Table 6). Two CV scenarios were considered, denoted as “across” and “within” families as also applied by [30]. In the across families approach, whole families were randomly assigned to training and testing sets, whereas in the within families approach, individuals from each family were randomized to training and testing sets. Subsequently, phenotypic predictions were performed using the three methods (BL, RKHS and BRANN) for both traits and for data sets containing either the full genotype set or subsets (201, 453 or 905 SNPs), with or without genotype imputation. Details on the imputation approach and models considered are provided below.

**Table 6:** **Number and distribution of individuals by trait and cross validation strategy employed**

Trait*	**Across families**		**Within families**		**Total no. of individuals**
	**Training set**	**Testing set**	**Training set**	**Testing set**	
BW	1,200	681	1,200	681	1,881
BMI	1,165	658	1,161	662	1,823

^*^BMI: body mass index; BW: body weight at ten weeks of age.

### Imputation

Testing sets containing 201, 453 and 905 SNPs were imputed to 1,809 SNPs using the Beagle software [38]. This software is based on Hidden Markov Models that cluster haplotypes at each locus. The clustering adapts to the amount of information available so that the number of clusters increases globally with sample size and locally with increasing linkage disequilibrium levels [14]. The training set, which contained 1,809 markers, was used as a reference sample for imputation of SNPs in the testing set. Imputation was carried out for both prediction scenarios (“across” and “within”) using only population structure and ignoring pedigree information. To check the global imputation accuracy, the imputed sets were compared with the full data set to calculate the percentage of correctly imputed genotypes.

### Bayesian LASSO

Tibshirani [39] proposed a regression method called Least Angle Shrinkage Selection Operator (LASSO) that combines feature subset selection and shrinkage estimation. In this model, a penalty term proportional to the norm of regression coefficients is added to the optimization problem formula, allowing for variable selection and shrinkage of coefficients simultaneously. The optimization problem can be expressed as:

$$ \underset{\beta }{ \min}\left\{{\displaystyle \sum_i{\left({y}_i-{\boldsymbol{x}}_{\mathrm{i}}\mathbf{\hbox{'}}\boldsymbol{\beta} \right)}^2}+\lambda {\displaystyle \sum_j\left\Vert {\beta}_j\right\Vert}\right\}, $$

where ${\displaystyle \sum_i{\left({y}_i-{\boldsymbol{x}}_{\mathrm{i}}\boldsymbol{\hbox{'}}\boldsymbol{\beta} \right)}^2}$ is the residual sum of squares and $\lambda {\displaystyle \sum_j\left\Vert {\beta}_j\right\Vert }$ is the penalization factor, with ***x***_***i***_ and ***β*** representing the incidence and parameter vectors, respectively, and *λ* is a regularization parameter. A larger *λ* means stronger shrinkage and some *β*’s are even zeroed out.

A Bayesian version of the LASSO was proposed by [40], who described a Gibbs sampling implementation. In this Bayesian interpretation, the LASSO solution can be viewed as a conditioned posterior mode in a Bayesian model with Gaussian likelihood, $p\left(\boldsymbol{y}\left|\boldsymbol{\beta}, {\sigma}_e^2\Big)={\displaystyle {\prod}_{i=1}^nN\Big({y}_i\left|{\boldsymbol{x}}_{\mathrm{i}}\boldsymbol{\hbox{'}}\boldsymbol{\beta}, \right.}\right.{\sigma}_e^2\right)$ and a conditional (given *λ*) prior on ***β*** that is a product of p independent, zero mean, double-exponential (DE) densities [40]. The double-exponential (or Laplace) distribution has a convenient hierarchical representation as a mixture of scaled Gaussian densities (e.g., [41]), i.e.:

$$ \begin{array}{l}{\beta}_j\sim DE\left({\beta}_j\Big|\lambda \right)=\frac{\lambda }{2}{e}^{-\lambda \left|{\beta}_j\right|}\\ {}={\displaystyle {\int}_0^{\infty}\left[\frac{1}{\sqrt{2\pi {\sigma}_j^2}}{e}^{-\left({\beta}_j^2/2{\sigma}_j^2\right)}\right]\left[\frac{\lambda^2}{2}{e}^{-{\lambda}^2/2{\sigma}_j^2}\right]}d{\sigma}_j^2.\end{array} $$

Convenient priors for the parameters of the Bayesian LASSO (BL) model have been suggested by [27] as:

$$ \begin{array}{l}p\left(\beta, {\sigma}_{\varepsilon}^2,{\tau}^2,{\lambda}^2\Big|H\right)=p\left(\boldsymbol{\beta} \left|{\sigma}_{\varepsilon}^2,{\boldsymbol{\tau}}^{\mathbf{2}}\left)p\right(\right.{\sigma}_{\varepsilon}^2\right)p\left({\tau}^2\left|\lambda \right.\right)p\left({\lambda}^2\left|{\alpha}_1,{\alpha}_2\right.\right)\\ {}=\left[{\displaystyle \prod_{j=1}^pN\Big({\beta}_j\left|0,{\tau}_j^2{\sigma}_{\varepsilon}^2\Big)\right.}\right]{\chi}^{-2}\left({\sigma}_{\varepsilon}^2\Big|d.f.,S\right)\\ {}x\left[{\displaystyle \prod_{j=1}^p \exp \left({\tau}_j^2\Big|\lambda \right)}\right]G\left({\lambda}^2\Big|{\alpha}_1,{\alpha}_2\right)\end{array} $$

where H is a set of hyper-parameters. Here, $p\Big(\boldsymbol{\beta} \left|{\sigma}_{\varepsilon}^2,{\boldsymbol{\tau}}^{\mathbf{2}}\Big)=\right.{\displaystyle \prod_{j=1}^pN\Big({\beta}_j\left|0,{\tau}_j^2{\sigma}_{\varepsilon}^2\Big)\right.}$ is the product of *p* normal densities with zero mean and variance ${\tau}_j^2{\sigma}_{\varepsilon}^2$ relative to each marker effect *j*. Further $p\left({\sigma}_{\varepsilon}^2\Big|d.f.,S\right)$ is a scaled inverted chi-square distribution ${\chi}^{-2}\left({\sigma}_{\varepsilon}^2\Big|d.f.,S\right)$ with *d.f*. degrees of freedom and scale parameter *S*; $\exp \left({\tau}_j^2\Big|\lambda \right)$ is an exponential distribution, and *p*(*λ*^2^|*α*_1_, *α*_2_) is a Gamma distribution with parameters *α*_1_ and *α*_2_. The parameter *λ*, also called smoothing parameter, plays a central role in the model as it controls the trade-off between goodness of fit and model complexity [39]. As its value approaches 0, the solution approximates a least squares solution; a large value of λ induces a sharper prior on β and, consequently, stronger shrinkage. Compared to Bayesian Ridge Regression, this model has the advantage of assigning a higher density to markers with zero effects, which seems biologically plausible [27].

The model was fitted to the training set in all scenarios considered. Inferences were based on a Gibbs sampling chain with 70,000 samples after a burn-in of 5,000. The parameters of the prior distribution were *S*_*ε*_ = *d. f*. _*ε*_ = *S*_*u*_ = *d. f*. _*u*_ = 1, and *α*_1_ =1.2 and *α*_2_ = 10^− 5^. The package BLR [42] developed for the R software was used for the analysis. Fitted models were then used to predict phenotypes in the testing set, and their predictive ability was assessed by the correlation between measured and predicted phenotypes, and by the PMSE.

### Reproducing Kernel Hilbert spaces regression

The RKHS theory was introduced by Aronszajn [43] and has been applied in statistics and machine learning (e.g., Support Vector Machines) fields for many years; foundations are provided in [44]. This semi-parametric approach was proposed by Gianola et al. [19,45] for regressing phenotypes on genotypes. The RKHS method has the property of having an infinite space of functions for searching the dependency between input and target variables, and the space is defined by the measure of distance used (in this case the type of kernel), without any additional assumptions on gene action or functional form. The method can be seen as a combination of the classical additive genetic model with an unknown function of markers, which is inferred nonparametrically, and has the potential of capturing complex interactions without explicitly modeling them [45]. To map the relationship between inputs (genotypes) and targets (phenotypes), a collection of functions defined in a Hilbert space (say *f* ∈ *H*) is used, from which an element, $\widehat{f}$, is chosen based on some criterion (e.g. penalized residual sum of squares or posterior density) [20]. The optimization problem for obtaining the estimates of RKHS is:

$$ \widehat{f}=\underset{f\in H}{ \arg \min}\left\{l\left(f,y\right)+\lambda {\left\Vert f\right\Vert}_H^2\right\}, $$

where *l*(*f*, *y*) is a loss function representing a measure of goodness of fit; ${\left\Vert f\right\Vert}_H^2$ is the squared norm of *f*, related to model complexity, and *λ* controls the trade-off between goodness of fit and model complexity.

According to the Moore-Aronszajn theorem [43], each RKHS is associated to a unique positive definite kernel. In RKHS, the markers are used to build a covariance or similarity matrix that measures distances between genotypes. Here, *Cov*(*g*_*i*_, *g*_*i* `_) ~ *K*(**x**_**i**_, **x**_**i** `_), with **x**_**i**_ and **x**_**i** `_ representing vectors containing genotypes for the *i*th and *i*’th individuals, and *K*(.,.) is the Reproducing Kernel (RK) related to a positive definite function [20].

The Kernel matrix (K) employed here was a Gaussian kernel, i.e. $K\left({\mathbf{x}}_{\mathbf{i}},{\mathbf{x}}_{\mathbf{i}\mathbf{\hbox{'}}}\right)= \exp \left\{-h\times {d}_{i{i}^{\hbox{'}}}\right\}$, where *h* is a bandwidth parameter and ${d}_{ii\hbox{'}}={\displaystyle {\sum}_{k=1}^p{\left({x}_{ik}-{x}_{i\hbox{'}k}\right)}^2}$ represents an element of the matrix of squared Euclidean distances among the individuals in the sample. The choice of *h* is a model selection issue and must consider the observed distribution of *d*_*ii*’_. In this study we used “kernel averaging” (multi-kernel fitting) as an automatic way of choosing the kernel based on the sample median of *d*_*ii*’_, as described by [46]. Hence, $h=a\times {q}_{0.5}^{-1}$ in which *a* was −5, −1 and −1/5, and *q*_0.5_ is the sample median of *d*_*ii*’_, for the three kernels used for kernel averaging. In this model, the genotypic values were the sum of three components, *g* = *f*_1_ + *f*_2_ + *f*_3_ , with $p\left({f}_1,{f}_2,{f}_3\left|{\sigma}_{\alpha, 1}^2\right.,{\sigma}_{\alpha, 2}^2,{\sigma}_{\alpha, 3}^2\right)=N\left({f}_1\left|0,{K}_1\right.{\sigma}_{\alpha, 1}^2\right)N\left({f}_2\left|0,{K}_2\right.{\sigma}_{\alpha, 2}^2\right)N\left({f}_3\left|0,{K}_3\right.{\sigma}_{\alpha, 3}^2\right)$. The variance parameters for these components were treated as unknown and assigned identical and independent scaled inverse chi-square prior distributions with degrees of freedom and scale parameters equal to *df* = *5* and *S* =(var(*y*)/2 × (*df* − 2)), respectively. Posterior distribution samples were obtained with a Gibbs sampler as described by de los Campos et al. [20]. Inferences were based on 50,000 samples after 5,000 samples of burn-in.

### Bayesian regularized artificial neural networks

A Bayesian Regularized Artificial Neural Network (BRANN) is a feed-forward network implemented with a maximum a posteriori approach in which the regularizer is the logarithm of the density of a prior distribution [47]. This model assigns a probability distribution to the network weights and biases, so that predictions are made in a Bayesian framework and generalization is improved over predictions made without Bayesian regularization. Details are in [48].

A basic feed-forward network uses initial weights and biases and transforms input information (in this case, genotype codes) through each given connected neuron in the hidden layer using an activation function. Information is then sent to the neuron in the output layer using another activation (transformation) function generating the output or predicted value. Next, the results are backpropagated (non-linear least-squares) in order to update weights and biases using derivatives. Therefore, no assumptions about the relationship between genotypes (input) and phenotypes (target) are made in this model. After training, outputs are calculated as:

$$ {\widehat{y}}_i=g\left\{{\displaystyle \sum_{k=1}^s{w}_kf\Big({\displaystyle \sum_{k=1}^R{w}_{k,i}{\underset{\sim }{x}}_i+{b}_k^l\Big)+{b}^2}}\right\}, $$

where *ŷ*_*i*_ is the predicted phenotype for an individual and ${\underset{\sim }{x}}_i$ are the input genotypes; *g* and *f* are the activation functions for output and hidden layers, respectively; *w*_*k*_ and *w*_*k*,*i*_ are the weights from neurons of the hidden to the output neuron, and from the input to the hidden neurons, respectively, and ${b}_k^1$ and *b*^2^ are the biases of the two layers. Training is the process by which the weights are modified in light of the data while the network attempts to produce an optimal outcome [25]. After training, the network can then be used to predict unknown phenotypes from individuals with genotype information.

In BRANN, in addition to the loss function given by the sum of squared errors, a penalty to large weights is also included in order to have a smoother mapping (regularization). The objective function is:

$$ f=\gamma {E}_D\left(D\Big|\underset{\sim }{w},M\right)+\alpha {E}_w\left(\underset{\sim }{w}\left|M\right.\right), $$

where $\left.{E}_D\Big(D\right|\underset{\sim }{w},M\Big)$ is the sum of squares of residuals in which *D* is the data (input data and target variable), $\underset{\sim }{w}$ are the weights and M is the architecture of the neural network. Further, ${E}_w\left(\underset{\sim }{w}\left|M\right.\right)$ is known as weight decay which is calculated as the sum of squares of weights of the network, and *α* and *γ* are the regularization parameters that control the trade-off between goodness of fit and smoothing.

The posterior distribution of *w* given *α*, *γ*, *D* and *M* is [49]:

$$ P\left(\left.w\right|D,\alpha, \gamma, M\right)=\frac{P\Big(D\left|w,\gamma, M\left)P\right(w\left|\alpha, M\Big)\right.\right.}{P\Big(D\left|\alpha, \gamma, M\Big)\right.}, $$

where *P*(*D*|*w*, *γ*, *M*) is the likelihood function, *P*(*w*|*α*, *M*) is the prior distribution on weights under the chosen architecture, and *P*(*D*|*α*, *γ*, *M*) is the normalization factor.

**Figure 1:**
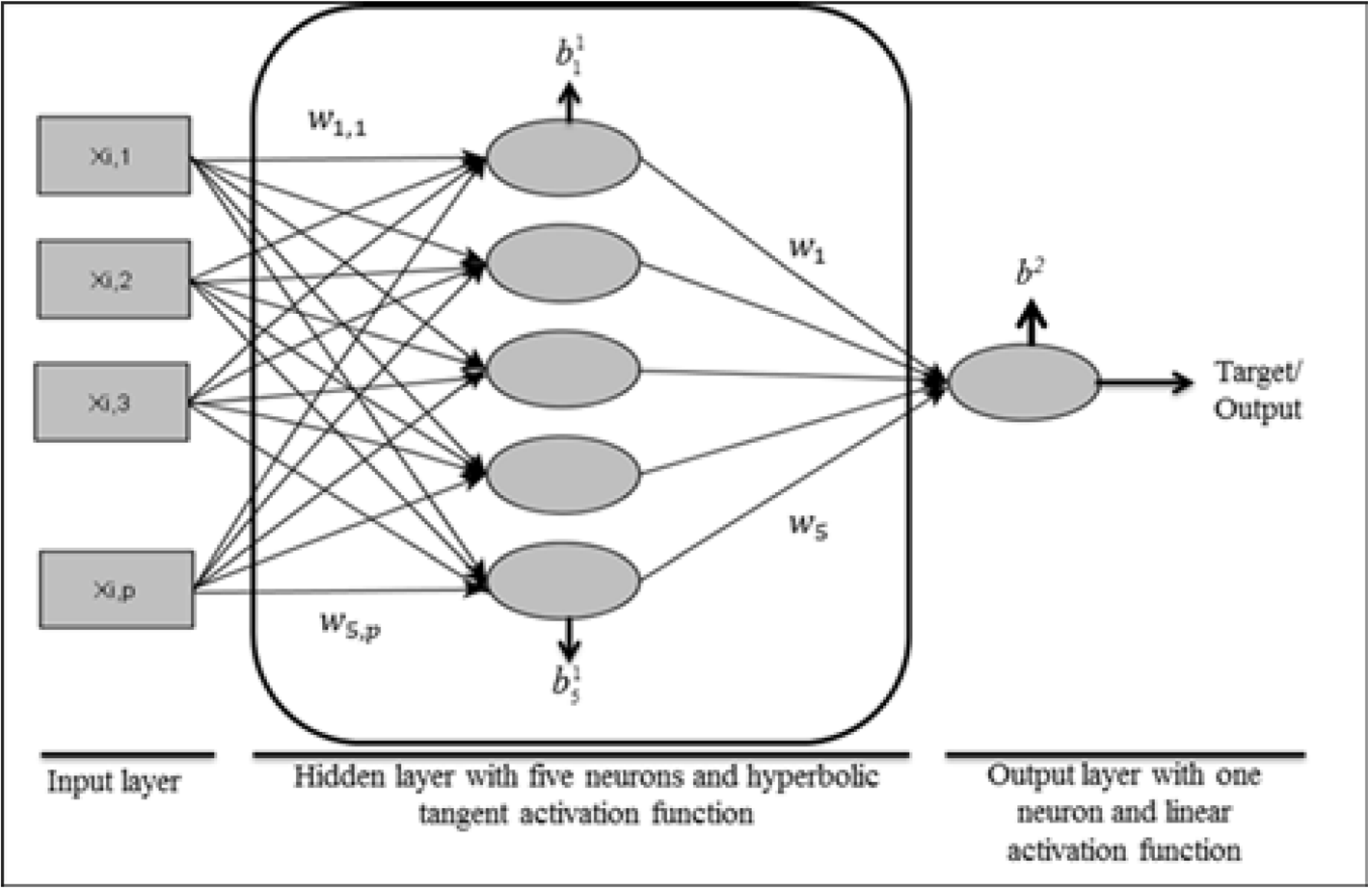
**Artificial Neural Network architecture with two layers containing 5 neurons in the hidden layer and one neuron in the output layer.** The *x*_*i*,*p*_ are the inputs for each animal *i*, and *p* is the number of SNPs; the *w*_*k*,,*j*_ are the weights where *k* is the hidden layer neuron indicator and *j* is the index for SNP; ${b}_k^l$ are the hidden layer biases, where *k* and l are the indexes for neurons and layers, respectively, and *b* ^*2*^ is the output neuron bias.

To assess overfitting, network architectures and number of epochs (iterations) were tested in a first step. A network containing 5 neurons in the hidden layer with a tangent sigmoid function and 1 neuron in the output layer with a linear function was used after such tests (Figure 1). The number of epochs was set to 30. Results were the average of 20 repetitions of the analysis with different randomly generated starting values. As an attempt to improve generalization, use of early stopping was tested for regularization, but Bayesian regularization worked better. The software MATLAB [50] was used for the analysis. The predictive ability was also assessed by correlation between estimated and measured phenotypes, and by PMSE, as it was for BL and RKHS.

### Availability of supporting data

The data set supporting the results of this article is available in the http://gscan.well.ox.ac.uk/.

## Abbreviations

BMI: Body mass index
BL: Bayesian LASSO
BRANN: Bayesian Regularized Artificial Neural Networks
BW: Body weight
CV: Cross-validation
LASSO: Least angle shrinkage selection operator
LD: Linkage disequilibrium
PMSE: Prediction mean squared error
RKHS: Reproducing kernel Hilbert spaces regression
SNP: Single nucleotide polymorphism
